# Morphing-Enabled Path Planning for Flying Tensegrity Robots as a Semidefinite Program

**DOI:** 10.3389/frobt.2022.812849

**Published:** 2022-03-14

**Authors:** Sergei Savin, Alexandr Klimchik

**Affiliations:** Robotics and Computer Vision Institute, Innopolis University, Innopolis, Russia

**Keywords:** tensegrity, deformation planning, path planning, semidefinite programming, bounding volumes

## Abstract

The development of deformable drones is of high importance but presents significant challenges. Such drones can be based on tensegrity structures, which leaves open the questions of configuration-space path planning for such robots. In this paper we propose a method that takes advantage of a simplified encoding of the drone’s shape, allowing to turn the path planning into a sequence of semidefinite programs. The mapping from the simplified description and the actual tensegrity configuration is done *via* a data-driven method, using a pre-computed dataset of statically stable configurations and their outer Löwner-John ellipsoids, as well as eigendecompositions of the ellipsoid matrices. Together it allows rapid containment check, whose computational cost depends linearly on the number of dataset entries. Thus, the proposed method offloads computationally-intensive parts to the offline dataset generation procedure, speeding up the algorithm execution.

## 1 Introduction

The last 2 decades have seen steady progress in the theory of tensegrity structures and their applications in Robotics. From tensegrity planetary landers [see Sabelhaus et al. (2015a); Vespignani et al. (2018)] to tensegrity spines for quadruped robots [Mirletz et al. (2014); Sabelhaus et al. (2015b); Zappetti et al. (2020)], the properties of these structures have provided new ways to design mobile objects performing difficult tasks. These properties include low weight, resistance to damage from collisions, the ability to control the stiffness of the structures, and the ability to control the deformation of the robot [see Liu et al. (2022) for a review of other properties of the tensegrity structures]. The latter properties motivate the interest in tensegrity as a structural element of soft robots [Lee et al. (2020); Rieffel and Mouret (2018); Kimber et al. (2019)].

One of the possible areas of application for tensegrity structures is deformable drones. Such robots can be used to traverse the cluttered environments, temporarily adjusting their shape to fit through narrow spaces, as well as take advantage of their higher maneuverability [Deng et al. (2020)]. Collision resilience should allow such drones to navigate partially uncertain environments, where exact positions and shapes of the obstacles are not always available. However, in order to effectively use such drones, two connected classes of problems applied to deformable tensegrity robots need to be solved: path planning and control. This paper is focused on the problems of the first class.

Same as in other areas of under-actuated robotics, path planning for deformable tensegrity structures requires finding a feasible path in the state space of the robot (assuming the environment is static, and the robot dynamics can be described by autonomous ODEs). And similar as in other high-dimensional systems, such as walking robots, a direct search for such paths is difficult; this makes simplified methods, based on conservative assumptions a favorable alternative. One of the most basic simplifications is the assumption that the trajectory of the robot is *quasi-static*, meaning that each configuration the robot assumes while traversing the path is statically stable (or there exists a control law that can make it become so). For a tensegrity structure, this means that each configuration it assumes should correspond to a local minimum of the potential energy of the structure. This allows us to transpose the path planning problem into a problem of finding a sequence of configurations that would path through the environment.

**FIGURE 1:**
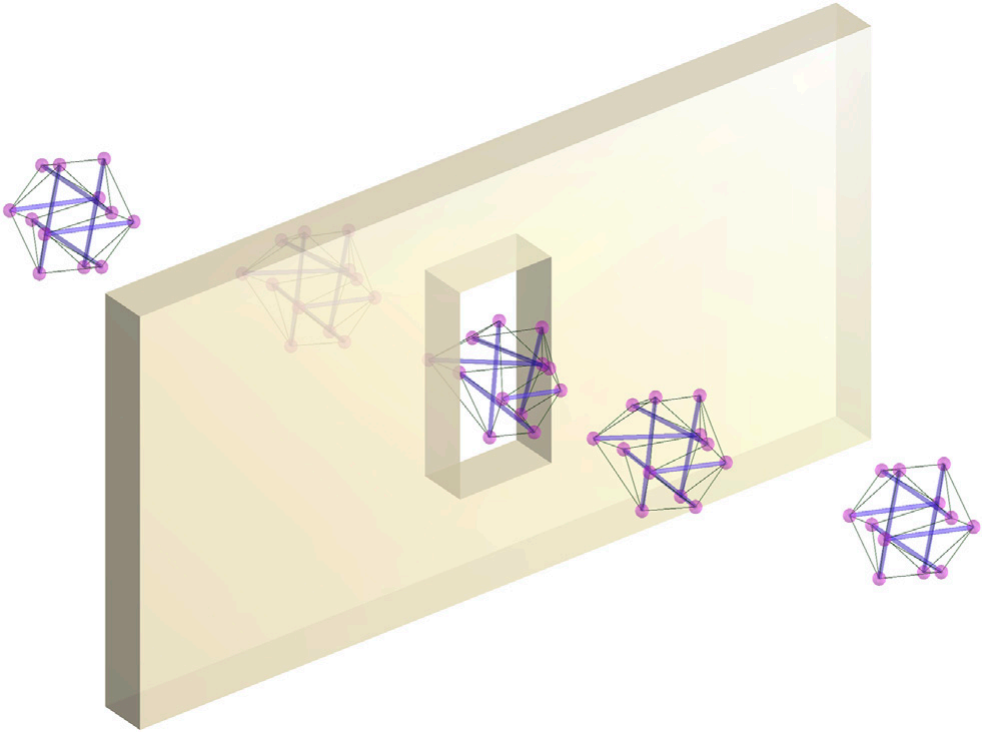
A deformable tensegrity drone changing its configuration to pass through a narrow window.

In this paper, we propose a method for finding sequences of configurations of deformable tensegrity drones as a sequence of deformations of the original shape, with the space of deformations being equivalent to the space of positive-definite matrices. This limits the possible shapes the structure can assume, but at the same time, it allows to cast the problem as a semi-definite program (SDP), taking advantage of the widely used solvers with well-understood properties. Moreover, the constraints in the proposed method have the form of linear-matrix inequalities (LMI), which is a familiar constraint type in a number of control-related applications. Figure 1 illustrates the work of the algorithm.

## 2 State of the Art

Motion planning for deformable objects is a wide field of study, with notable sub-fields. For example, motion planning for rod-like structures (generalized under the category of deformable linear objects) has been well studied [see Moll and Kavraki (2006); Roussel et al. (2014); Shah and Shah (2016)]; there, such structures are usually manipulated by external forces. Similarly, methods for manipulation-based motion planning for objects of other shapes have been proposed Anshelevich et al. (2000). Here we concentrate on the case when the robot uses internal resources for deformation. This requires a way to predict the form of the structure, given the distribution of forces and torques acting on it.

As was discussed previously, limiting ourselves to the quasi-static case (when the speeds and accelerations of the motion are such that the inertial forces do not play a significant role in the robot’s dynamics) allows us to cast the problem of finding the shape of the structure assumes under the given distribution of forces as a problem of finding static equilibrium. For fixed rest lengths of the elastic elements that form tensegrity structure, the static equilibrium problem can be solved by minimizing potential energy of the structure, or equivalently, by solving a feasibility problem, given force equilibrium equations and force limits arising from the physical properties of the elements of which the structure consists: the cables can only experience tensile forces, while the struts are designed to experience compressive forces. Both result in nonlinear optimization problems. Fixing positions of the nodes, the feasibility problem can be solved as a single convex program, using force density variables [see Xu et al. (2018); Khafizov and Savin (2021)]. A more general form of this problem, known as form-finding, is discussed in a number of publications, including Tibert and Pellegrino (2003); Masic et al. (2005); Zhang et al. (2006).

Alternatively, data-driven and learning-based methods can be used for the same end. It was shown that a neural network can be used to predict the statically-stable configuration of the tensegrity structure, given the rest lengths of its elastic elements [see Zalyaev et al. (2020)]. Similarly, evolutionary methods for the design of statically stable structures have been proposed Paul et al. (2005). Note that the latter is close to topological tensegrity design methods based on force density variables, as presented in Xu et al. (2018); Koohestani (2020), as they focus on the design of the structures, rather than finding the static equilibrium of the existing ones. We note that algorithms mentioned in this paragraph are either limited to the specific types of tensegrity robots, or are computationally intensive, requiring the solution of optimization problems, or running neural networks.

One of the many challenges associated with motion planning for deformable robots is finding paths that require feasible deformations Mahoney et al. (2010). Simplified models of the deformable objects are an essential tool in motion planning for such robots Mahoney et al. (2010). In Gayle et al. (2005), deformable robot was modelled as an elastic system. One of the methods proposed in Bayazit et al. (2002) uses bounding box deformation to correct the shape of the robot. Here we propose to encode deformations with as positive-definite matrices, thus limiting possible deformations, but making the space of all considered deformations convex.

Taking advantage of the simplified encoding described above we propose a data-driven ellipsoid mapping, which allows us to rapidly search for tensegrity configurations compatible with the requested deformation, lying inside the requested ellipsoids. This is done by pre-computing covering Löwner-John ellipsoids and the use of eigendecomposition of these ellipsoids, which allows checking ellipsoid containment based on algebraic operations.

The rest of the paper is organized as follows. Section 3 describes static equilibrium conditions for the structure. Section 4 discusses the use of Löwner-John ellipsoids as bounding volumes, presents optimization problems associated with computing these ellipsoids, and gives forms of self-intersection problem for the structure; all three topics form the basis for dataset generation algorithm and the mapping method, presented in Section 5. Section 6 presents motion planning based on the proposed method. The computational cost associated with the proposed algorithm is given in Section 8.

## 3 Static Equilibrium of Tensegrity Structures

The main elements making a tensegrity structure are cables and struts, with cables experiencing purely tensile forces, and struts experiencing purely compressive forces. Here we focus specifically on a sub-class of tensegrity structures where no two struts are immediately connected; this is the class of tensegrity structures that have been the focus of the majority of the tensegrity-related research, due to the comparative ease of design of such structures.

The connection points between a strut and cables are called nodes. One of the ways to describe a configuration of a tensegrity structure is to describe the position of its nodes. Here we denote the position of the nodes by vector variables **r**_*i*_.

### 3.1 Static Equilibrium Conditions

In order for the structure to maintain static equilibrium, the sum of forces acting on each node should be zero. Denoting a force acting on the node **r**_*i*_ from the node **r**_*j*_ as **f**_*i*,*j*_, this condition can be written as:

$$\overset{n}{\underset{j=1}{\sum }}f_{ij}\left(r_{i},r_{j}\right)=0,\;\forall i$$ (1)

where *n* is the total number of nodes in tensegrity structure. Assuming that both the struts and the cables can be modelled as linear springs, the forces **f**_*i*,*j*_ acting between two nodes can be modelled as follows:

$$f_{i,j}=C_{i,j}\mu_{i,j}\left(\|r_{i}-r_{j}\|-\rho_{i,j}\right)\frac{r_{i}-r_{j}}{\left\|r_{i}-r_{j}\right\|}.$$ (2)

where *μ*_*i*,*j*_ is the stiffness coefficient, *ρ*_*i*,*j*_ is the rest length of the given elastic element (distance between the nodes when the force **f**_*i*,*j*_ equals zero) and *C*_*i*,*j*_ ∈ {0, 1} is the connectivity identifier and is equal to zero if no connection exists between the two elements.

### 3.2 Stable Configuration Problem

Assuming that stiffness coefficients *μ*_*i*,*j*_ are given and constant, but node positions **r**_*i*_ are functions of the rest lengths *ρ*_*i*,*j*_, we can formulate the direct stable configuration problem: given *ρ*_*i*,*j*_, find **r**_*i*_ such that the system is in static equilibrium, and the inverse stable configuration problem: given **r**_*i*_, find such *ρ*_*i*,*j*_ that the system is in static equilibrium. Denoting vector of all parameters **r**_*i*_ as **r** and vector of all parameters *ρ*_*i*,*j*_ as *ρ*, we can define functions DFF(⋅), IFF(⋅) solving direct and inverse stable configuration problems:

$$r=\text{DFF}\left(\rho \right),\;\;\;\rho =\text{IFF}\left(r\right).$$ (3)

Multiple algorithms implementing functions (3) can be found in the literature. However, all implementations share a number of properties.

Firstly, we note that for tensegrity structures not in contact with the environment, simultaneous translations of all nodes do not influence the static equilibrium conditions. Thus, there are infinitely many solutions to direct stable configuration problem. Moreover, since a stable equilibrium configuration with a non-degenerate stiffness matrix corresponds to a local minimum of the potential energy of the structure, a small change in *ρ*_*i*,*j*_ corresponds to a small shift in that local minimum, implying the existence of the solution to the perturbed problem.

Secondly, we note that for practical tensegrity structures [including tensegrity prism and 6-bar tensegrity structure, see Vespignani et al. (2018)] variables **r** span a higher dimensional space than *ρ*; this implies that there are configurations that cannot be stabilized, i.e., the inverse problem does not always have a solution.

## 4 Simplified Descriptions of the Robot and the Environment

In order to make the motion planning problem tractable, both the robot and the environment need to be described in a form that facilitates efficient computations. To this end we describe the environment as a collection of convex polytopic obstacle-free spaces $S_{p}$ , given as H-polytopes: $S_{p}=\left\{r:\;S_{p}r\leq h_{p}\right\}$ . In the following subsections, we demonstrate how a deformable drone shape can be represented, and how this representation interacts with the obstacle-free spaces.

### 4.1 Bounding Volume for a Tensegrity Structure

**FIGURE 2:**
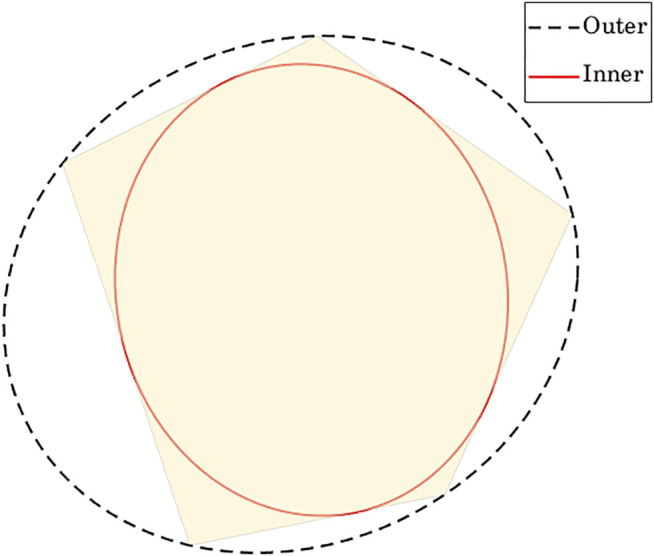
A polytope with inner and outer Löwner-John ellipsoids.

The method proposed in this paper requires the use of bounding volumes. Both bounding boxes and bounding ellipsoids can be used, but we limit our discussion to bounding ellipsoids. We use outer Löwner-John ellipsoid (the smallest volume ellipsoid that contains a given set of points) as a bounding volume containing all nodes of the tensegrity structure; Figure 2 illustrates the geometry of Löwner-John ellipsoids.

Finding outer Löwner-John ellipsoid containing all nodes **r**_*i*_ can be cast as a convex optimization [see Boyd et al. (2004)]:

$$\begin{array}{cccc} & \underset{Y,\;y}{\text{minimize}} & & \text{log det }Y^{-1},\\ & \text{subject to} & & \|Yr_{i}+y\|\leq 1,\;\forall i, \end{array}$$ (4)

where **Y** is positive semidefinite ellipsoid deformation matrix and **y** is ellipsoid center. Resulting ellipsoid E in set notation is given as E={r:‖Yr+y‖≤1} . In practice, minimization of log det **Y** ^−1^ is often replaced by maximization of det (**Y**)^1/*n*^ function.

### 4.2 Free-Space Containment

We need to be able to check if the ellipsoid representing the outer approximation of the tensegrity structure fits inside a given obstacle-free region S . To this end, it is more convenient to use a dual ellipsoid representation E={Xr+x:‖r‖≤1} . The connection with the primal representation is established by the following relations: **X** = **Y** ^−1^, **x** = − **Y** ^−1^ **y**. We do not attempt to find the dual representation of the outer Löwner-John ellipsoid as a single optimization problem; instead, we find the primal representation of the ellipsoid, and then convert it when needed.

Given an obstacle-free region $S_{p}=\left\{r:\;S_{p}r\leq h_{p}\right\}$ , where $S_{p}={\left[\begin{array}{ccc} s_{1,p} & \ldots & s_{m,p} \end{array}\right]}^{⊤}$ , with **s**_*j*,*p*_ forming rows of the matrix **S**_*p*_ in the H-representation of the obstacle-free region and $h_{p}={\left[\begin{array}{ccc} h_{1,p} & \ldots & h_{m,p} \end{array}\right]}^{⊤}$ , finding inner Löwner-John ellipsoid in dual representation contained in $S_{p}$ can be written as the following optimization problem [see Boyd et al. (2004) for the derivation]:

$$\begin{array}{cccc} & \underset{X,\;x}{\text{minimize}} & & \text{log det }X^{-1},\\ & \text{subject to} & & \|Xs_{j,p}\|+s_{j,p}^{⊤}x\leq h_{j,p},\;\forall j \end{array}$$ (5)

### 4.3 Self-Intersection

One of the typical problems for tensegrity structures is self-collisions and self-intersections. While self-collisions do not pose practical problems during the motion of the robot, they need to be taken into account when a configuration-space trajectory for such a robot is planned.

Assume a pair of nodes **r**_1_ and **r**_2_ is connected via a cable or a rod, and so is a pair of nodes **r**_3_ and **r**_4_. In order to check if these two structural elements (rods or cables) of the structure intersect in a given configuration, we can solve the following linear problem:

$$\begin{array}{cccc} & \text{find} & & z,\\ & \text{subject to} & & \left\{\begin{array}{c} \left[\begin{array}{cc} \left(r_{1}-r_{2}\right) & \left(r_{3}-r_{4}\right) \end{array}\right]z=r_{4}-r_{2}\;\\ z\geq 0\;\\ \|z{\|}_{\infty }\leq 1,\; \end{array}\right. \end{array}$$ (6)

where **z** determines the position of the intersection point with respect to the nodes **r**_1_, **r**_2_, **r**_3_ and **r**_4_. If the problem (6) has a solution, there is an intersection between the two structural elements. In order to explicitly control the precision of the algorithm, we can add numeric tolerance threshold *δ*_*in*_:

$$\begin{array}{cccc} & \text{find} & & z,\\ & \text{subject to} & & \left\{\begin{array}{c} {\left|\left|\left[\begin{array}{cc} \left(r_{1}-r_{2}\right) & \left(r_{3}-r_{4}\right) \end{array}\right]z-r_{4}+r_{2}\right|\right|}_{2}\leq \delta_{in}\;\\ z\geq 0\;\\ \|z{\|}_{\infty }\leq 1,\; \end{array}\right. \end{array}$$ (7)

The functions (6) and (7) can be useful in numeric dataset generation procedures, as will be shown in the next section.

### 4.4 Cable Slack

Another problem of tensegrity structures is cable slack. While it does not pose immediate problems by itself (as long as the stiffness matrix of the structure does not become degenerate), it may still lead to a more complex behaviour of the system. With that in mind we would like to avoid slackened cables.

One of the simplest ways to encode the taut cables constraint is to require that the current length of cables exceeds their rest length by a given margin *l*_*m*_:

$$C_{i,j}\left(\|r_{i}-r_{j}\|-\rho_{i,j}-l_{m}\right)\geq 0,\;\;\forall i,j.$$ (8)

We denote this constraint as slack(**r**) further in the text.

## 5 Mapping Ellipsoids to Configurations

### 5.1 Configuration Dataset

In order to map an ellipsoid to an actual robot configuration, we use a configuration dataset. Each configuration in the dataset is given by two sets of parameters: **r** and *ρ*. There are two principal ways of obtaining the dataset: 1) collecting the data on statically stable configurations of a given tensegrity structure (*via* a motion capture system, for example), while measuring or estimating rest lengths of the elastic elements *ρ*, or 2) generating a number of sets of rest lengths *ρ* and solving the direct stable configuration problem. Here we use the second approach. Its main advantage is that it allows us to easily change the dataset, as the generation process involves solving a pair of small-size quadratic programs; its downside is the necessity to detect and discard infeasible configurations. The following subsections will elaborate on this last point.

Our process of generating the dataset involves the following steps. On the first step, a base rest length vector *ρ*_*b*_ is proposed. We will consider the statically stable configuration **r**_*b*_ = DFF(*ρ*_*b*_), associated with *ρ*_*b*_, as undeformed. On the second step, an array of perturbation directions *ρ*_*d*,1_, … , *ρ*_*d*,*N*_ is generated, where *N* is the number of directions in the dataset; we sampled *ρ*_*d*,*i*_ it from a uniform distribution. For each direction *ρ*_*d*,*i*_ we solve the direct form finding problem **r** = DFF(*ρ*_*b*_ + *αρ*_*d*,*i*_), moving the value of alpha from 0 to 1. For each configuration thus obtained we check self-intersection of the structural elements (rods and cables), using expression (7); if intersection did occur, the configuration is not stored, and no further configurations associated with this direction are computed. If no configuration occurred, the configuration **r** and rest length vector *ρ* = *ρ*_*b*_ + *αρ*_*d*,*i*_ are both stored in the dataset.

We augment the dataset with two additional fields that are used in the following sections: perturbation norm and eigendecomposition of the outer Löwner-John ellipsoid. We define the perturbation norm *ν* as the 2-norm of the perturbation Δ*ρ*: *ν* = ‖Δ*ρ*‖. It will later be used as a deformation measure.

In order to find the outer Löwner-John ellipsoid for the given configurations **r**, we solve problem (4), obtaining corresponding matrix **Y**. Then we find the following eigendecomposition:

$$Y^{-1}V=VD$$ (9)

Thus found values of Δ*ρ*, **r**, *ν*, **V** and **D** form the dataset. The process of compiling the dataset is outlined as Algorithm 1, where Intersection (⋅) checks if (7) is feasible, Löwner-John (⋅) solves (4), and eigendecomposition (⋅) returns an eigendecomposition of a matrix. Note that the perturbation norm *ν* is augmented with a regularization term *ξ* det (**Y** ^−1^), which aims at punishing configurations that result in low volume ellipsoids; *ξ* is a scalar regularization coefficient.

Algorithm 1 Dataset generation algorithm.

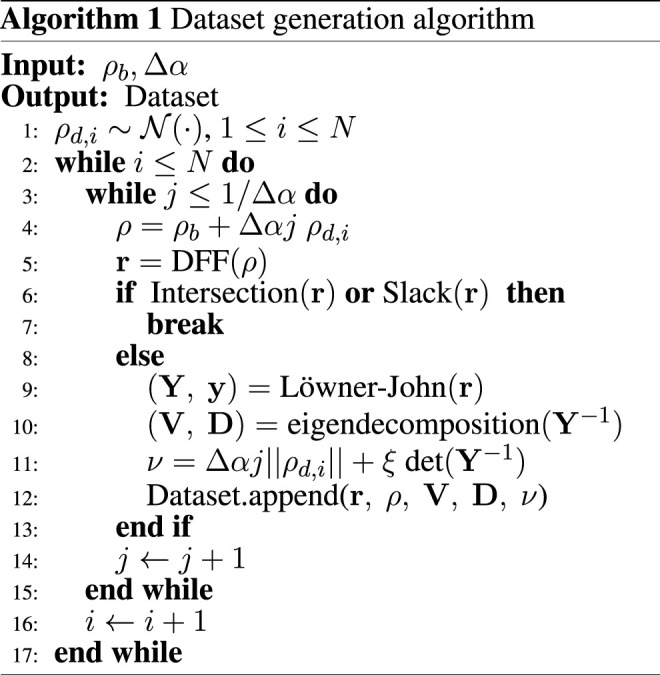

### 5.2 Dataset-Based Configuration Mapping

Given an ellipsoid in dual representation E={Xr+x:‖r‖≤1} , we can rapidly check which of the configurations from the dataset can be contained in the ellipsoid E . To do it we define containment matrix **W**:

$$W=X^{-1}VD$$ (10)

With that we can assign a measure to each configuration, using either a step function or a log barrier. The expression for the later is given below:

$$v=\nu +\phi \left(1-e_{1}^{⊤}W^{⊤}We_{1}\right)+\phi \left(1-e_{2}^{⊤}W^{⊤}We_{2}\right)+\phi \left(1-e_{3}^{⊤}W^{⊤}We_{3}\right),$$ (11)

$$\left\{\begin{array}{cc} \phi \left(x\right)=-\delta \text{log}\left(x\right)\; & \text{if}\;\;\;x>0,\\ v=\infty \; & \text{otherwise}, \end{array}\right.$$ (12)

where $e_{1}={\left[\begin{array}{ccc} 1 & 0 & 0 \end{array}\right]}^{⊤}$ , $e_{2}={\left[\begin{array}{ccc} 0 & 1 & 0 \end{array}\right]}^{⊤}$ , and $e_{3}={\left[\begin{array}{ccc} 0 & 0 & 1 \end{array}\right]}^{⊤}$ are unit vectors forming axes of the world coordinate frame.

Then, finding the configuration that corresponds to the smallest *v* we find the desired map. Note that the map is not designed to find the closest possible configuration to the given ellipsoid, and instead it tries to find the one with the least deformation that can fit in it.

### 5.3 Drone-Specific Considerations

The use of a tensegrity structure as a frame of a drone entails a large number of problems. This paper does not aim to present a comprehensive study or give solutions to these problems; however, we can list a few important considerations connected with the proposed dataset generation method that need to be addressed when using the method for planning a sequence of deformation for a tensegrity drone.

Firstly, depending on the number of motors and on how they are attached and oriented, some configurations of the tensegrity structure might be impossible to stabilize in the air. These should be identified and removed. The process of identifying these configurations depends both on the position of the motors and the control algorithm designed for the drone.

Secondly, the geometry of the propellers needs to be taken into account when the configuration of the tensegrity is being evaluated as admissible or not. If the volume covered by the propellers can be approximated as a convex polytope, and if the position of this volume can be approximated as a linear form of the positions of the nodes of the structure, then the problem of finding self-intersections involving the propellers can also be cast as a quadratic problem. The same can be said about other structural elements of the drone: the batteries, the controller and transmission boards, etc.

## 6 Quasi-Static Deformation-Enabled Motion Planning

The goal of the quasi-static deformation-enabled motion planning is to provide a sequence of positions and deformations of the robot, as it moves from the initial to the goal point, such that no constraints are violated. Here we only consider constraints associated with containment in the obstacle-free regions.

### 6.1 Obstacle-free Region Intersections

To simplify computations we make a conservative assumption that in order for the robot to be able to pass from one region to another, it has to be able to fit in the intersection of the two regions. This assumption can be prohibitive if the free space is tessellated into non-intersecting obstacle-free regions. However, it can be made less conservative if maximum-volume convex obstacle-free regions are generated from seed points [as was done in Deits and Tedrake (2015); Savin (2017)], and the free space is densely populated by the seed points.

Note that the requirement that two regions have a sufficiently large intersection in order for the robot to be able to pass from one to the other allows treating the obstacle-free regions as graph edges, and intersections as graph vertices. Therefore a path from the region containing the initial point to the region containing the goal point is also a path along this graph. Thus the path can be found by graph search methods, such as A* algorithm. The heuristic for the search can be based on the distances between the centers of the region intersections that share a region, as well as the width of the region; both can be approximated by finding the inner Löwner-John ellipsoid (5), for the intersection of the regions with **x** giving the ellipsoid’s center acting as an approximation of the region’s center, and the width of the ellipsoid acting as an approximation of the region’s width.

**FIGURE 3:**
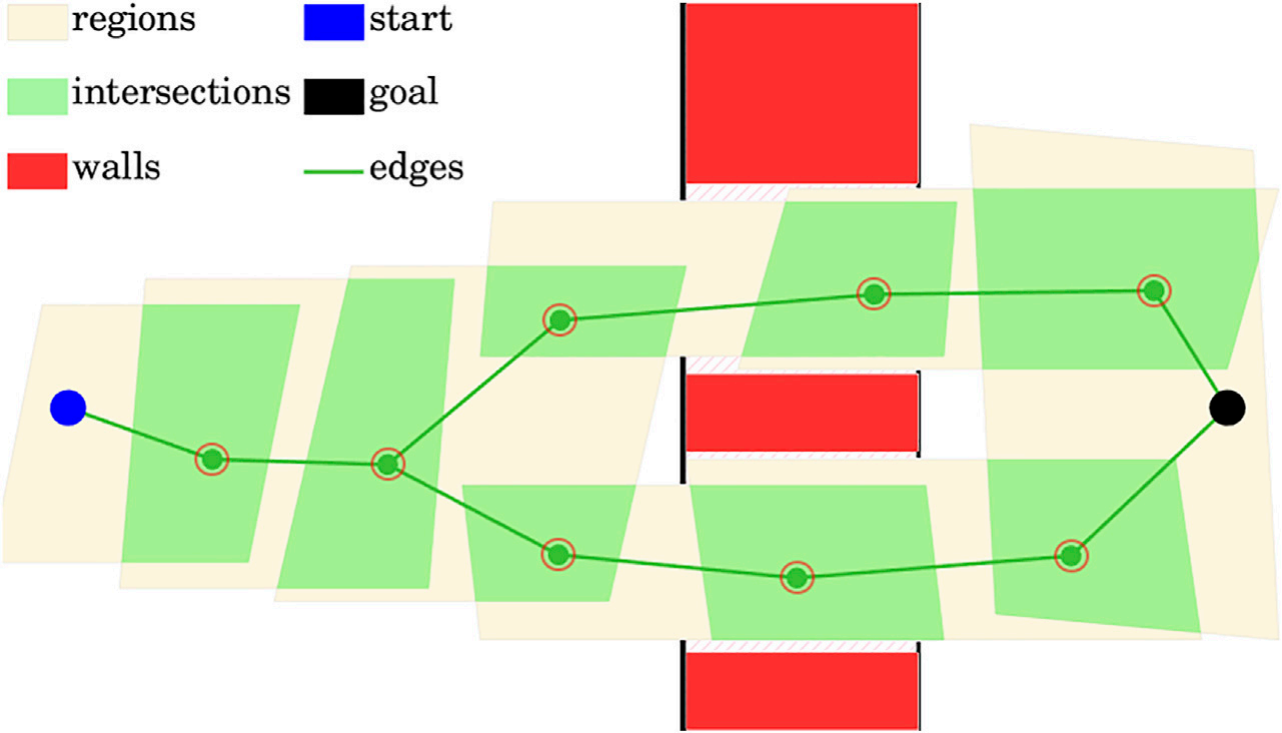
Graph representation of the path, based on obstacle-free region intersections.

Figure 3 illustrates the graph representation of the path, based on obstacle-free region intersections. The graph nodes and graph edges are shown, generating at least two paths from the starting point to the goal point, passing through different openings in the wall (its intersection shown in red). Note that on the picture the obstacle-free space is not densely populated with large obstacle-free regions, and hence their intersections are unnecessarily small, illustrating the conservativeness of the requirement in this case.

### 6.2 Inflation-Based Deformation Planning

In this subsection we assume that a single path from the initial point to the goal point is given in form of a sequence of regions $S_{1},\;\ldots S_{n}$ , and the robot needs to pass through intersections of all consecutive regions in that sequence. The proposed inflation-based deformation planning methods consists of two steps: 1) find the biggest possible ellipsoid that fits in each region and in each intersection that needs to be passed, 2) for each ellipsoid, find a robot configuration that can fit in it with the *least deformation* *v*.

To check if the ellipsoid simultaneously lies in two regions $S_{p}=\left\{r:\;S_{p}r\leq h_{p}\right\}$ and $S_{p+1}=\left\{r:\;S_{p+1}r\leq h_{p+1}\right\}$ , where $S_{p}={\left[\begin{array}{ccc} s_{1,p} & \ldots & s_{m_{1},p} \end{array}\right]}^{⊤}\in R^{m_{1}\times 3}$ and $S_{p+1}={\left[\begin{array}{ccc} s_{1,p+1} & \ldots & s_{m_{2},p+1} \end{array}\right]}^{⊤}\in R^{m_{2}\times 3}$ , it is sufficient to check if it lies in their intersection, whose H-representation is constructed by concatenation: $S_{p}\cap S_{p+1}=\left\{r:\;{\left[\begin{array}{cc} S_{p}^{⊤} & S_{p+1}^{⊤} \end{array}\right]}^{⊤}r\leq {\left[\begin{array}{cc} h_{p}^{⊤} & h_{p+1}^{⊤} \end{array}\right]}^{⊤}\right\}$ . The problem (5) then takes form:

$$\begin{array}{cccc} & \underset{X_{p,p+1},\;x_{p,p+1}}{\text{minimize}} & & \text{log det }X_{p,p+1}^{-1},\\ & \text{subject to} & & \left\{\begin{array}{cc} \|X_{p,p+1}s_{j,p}\|+s_{j,p}^{⊤}x_{p,p+1}\leq h_{j,p},\; & 1\leq j\leq m_{1},\\ \|X_{p,p+1}s_{k,p+1}\|+s_{k,p+1}^{⊤}x_{p,p+1}\leq h_{k,p+1},\; & 1\leq k\leq m_{2}. \end{array}\right. \end{array}$$ (13)

Note that problem (5) can be solved independently for each region $S_{p}$ and each region intersection $S_{p}\cap S_{p+1}$ . This alleviates numerical problems associated with the growth in the number of variables in the planning problem.

The second step of the process consists in mapping found ellipsoids to configurations from the dataset. On this step we go through found ellipsoids *E*_*p*_ = {**X**_*p*_ **r** + **x**_*p*_: ‖**r**‖ ≤ 1} and *E*_*p*,*p*+1_ = {**X**_*p*,*p*+1_ **r** + **x**_*p*,*p*+1_: ‖**r**‖ ≤ 1} sequentially, and for each of them we use (10) and (11) to determine the deformation value *v* of each configuration in the dataset; the configuration with the smallest *v* is chosen as the image of the respective ellipsoid. This process is described in the Algorithm 2.

Algorithm 2 Ellipsoids mapping.

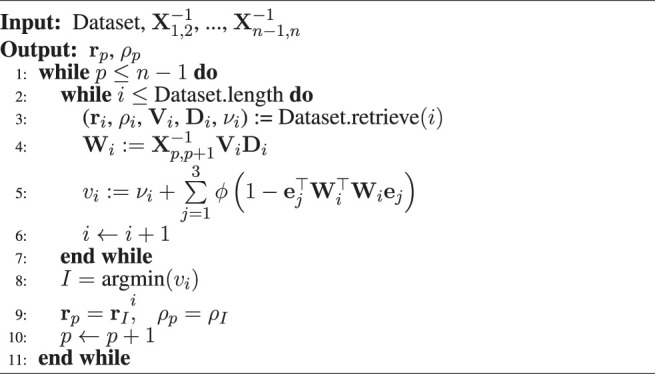

The described above algorithm can be used for planning deformations of a drone, as it passes through narrow spaces. However, let us note that the algorithm only provides the desired deformations (and corresponding node positions and cable rest lengths) for a few trajectory nodes; hence, the evolution of the nodes and the evolution of the cable rest lengths need to be determined separately, e.g., *via* interpolation. With that in mind it is of further interest to study resulting trajectories. Such a study is presented in the following section.

Additionally, let us remark that if the additional safety margins are required, the ellipsoids representing obstacle-free regions can be scaled down by a given amount. Alternatively, the required safety margins can be checked directly on the configurations found by the algorithm; this might be preferable, as the use of ellipsoids to approximate obstacle-free spaces already makes the algorithm conservative.

## 7 Simulation Results

In this section, we provide simulation results, which illustrate the work of the algorithm. As was mentioned in the last section, the algorithm is providing a sequence of deformations, but between each pair of consecutive deformations, the rest lengths of the elastic elements should be determined separately. Here we take the simplest case when the linear interpolation is used. We study the resulting trajectories in terms of the mechanical properties of the structure as it undergoes deformation.

**FIGURE 4:**
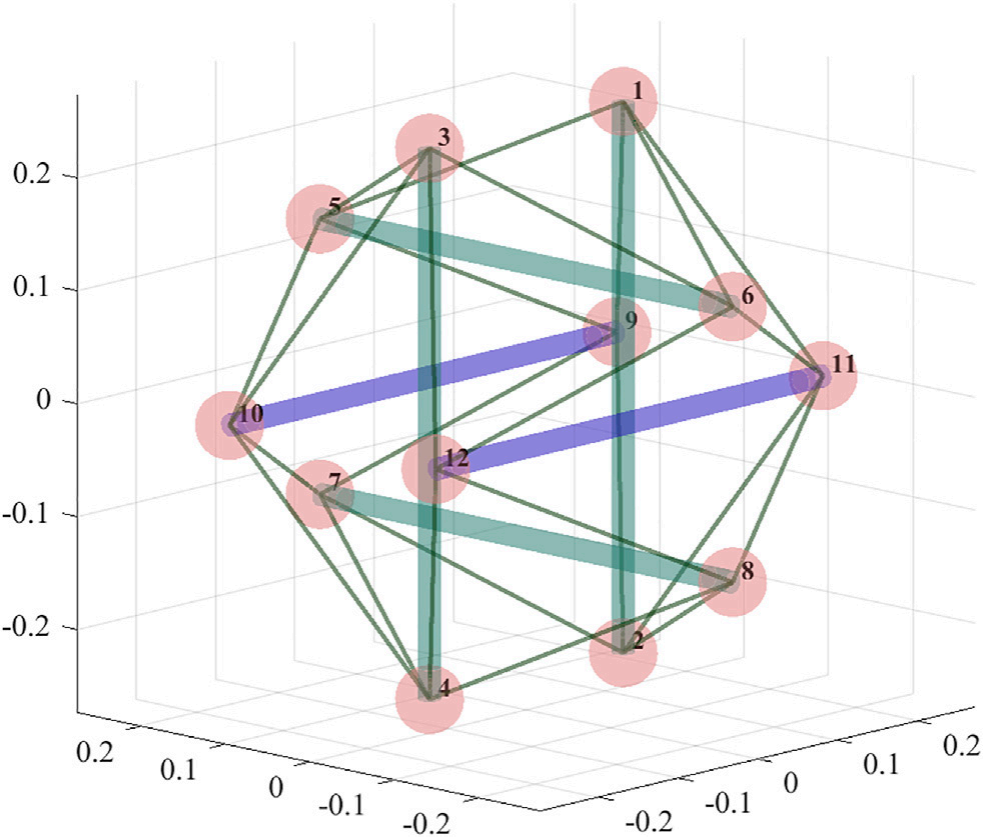
A 6-bar tensegrity structure with two actuated rods (rods that can control their rest lengths), shown in blue; nodes of the structure are numbered.

In order to provide a higher range of deformation, while maintaining the orientation of the rods (which is preferable for a drone where rotors are rigidly attached to the rods) we choose to actuate symmetrically placed rods. In Figure 4 the actuated rods are shown in blue, while the unactuated cables and rods are shown in green. Actuated rods connect nodes 9 and 10, and nodes 11 and 12, as shown in Figure 4.

Note that the practicality of such actuation pattern depends on the ability of the drone to effectively change and maintain its orientation; if the drone can be relied on to maneuver itself in such a way as to face the narrow opening with the actuated horizontal rods being orthogonal to the drone trajectory as it traverses the opening, then the actuation pattern is justified, as the other pair of horizontal rods would be oriented tangentially to the trajectory, not influencing the ability of the robot to fit through the opening. If the opening is horizontal rather than vertical (wide and short rather than narrow and toll), the vertical rods would also require to be actuated. In this section, we consider the simpler case for a cleaner presentation.

We consider the case when the structure has to pass through a tall opening with a width of 0.35 m. The undeformed width of the structure is 0.488 m. Two possible concerns with the resulting trajectory are 1) cable slack, and 2) excessive tension in some of the cables.

**FIGURE 5:**
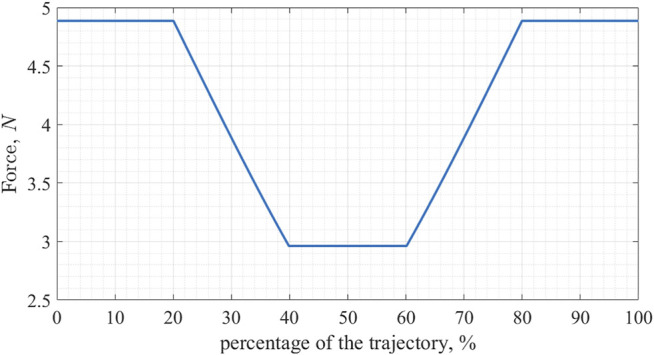
Evolution of the magnitude of tensile forces for two cables; cable 1 connects nodes 10 and 3, and cable 2 connects nodes 8 and 12.

Firstly, we study the evolution of the tensile forces in the structure’s cables. As a representative example we choose the cable between nodes 10 and 3 (connecting one of the actuated rods with one of the vertical ones) denoted as cable 1, and a cable between nodes 8 and 12, denoted as cable 2, using the notation in Figure 4. Figure 5 shows the evolution of the tensile forces in the cables along the trajectory. The first 20% of the trajectory the structure maintains its shape, next 20% it deforms, then it maintains the deformed shape (during which period the structure is required to pass through the opening), then the structure deforms back into its original shape and maintains it until the end of the trajectory. Note that the timing is chosen arbitrarily; since the deformation planning requires purely geometric information, the timing of the trajectory can be planned separately taking into account its dynamical properties.

As we can see in Figure 5 the tensile force in the cable goes down during the deformation, and then back up afterward. At its lowest point, it goes down by 39.4% for the cable 1 and by 60% for the cable 2. With the trajectory planned for this experiment, no cable loses more than 60% of its initial tensile force value, and only cables connecting nodes 6, 8, 11, and 12 do so.

We should note that while in this experiment the tensile forces were not increasing throughout the motion and the relative decrease of these forces was limited to 60% of their initial values, the dataset used in generating the trajectory does include a higher range of force changes. Overall in the dataset of 200 elements, the highest recorded tensile force exceeds the nominal value by 55.9%, while the smallest is only 4.78% of the nominal value. Whether or not these values can lead to mechanical damage of the structure, the loaded elements, or the cables themselves depends on a choice of materials and the design of mechanical parts (e.g., connectors between the cables and struts).

Presented here study can serve as an illustration of how the analysis of the trajectory quality can be done in practice. In our case we can be satisfied that the structure is capable of following the found trajectory since the tensile forces are clearly within the nominal range, but for arbitrary trajectories generated with the same dataset, we need to check the ability of the cables to withstand a 55.9% increase in the tensile force magnitudes. If such an increase of tensile force magnitude is undesirable, the corresponding elements can be removed from the dataset, or a re-design of the cables can be suggested.

## 8 Computational Cost

In order to assess the computational cost of the algorithm, we need to recognize that the number of optimization programs (13) to be executed is equal to the number of intersections in the path. The number of variables in the problem formulation is independent of the problem geometry, but the number of constraints can change, subject to the shape of the region intersections.

**FIGURE 6:**
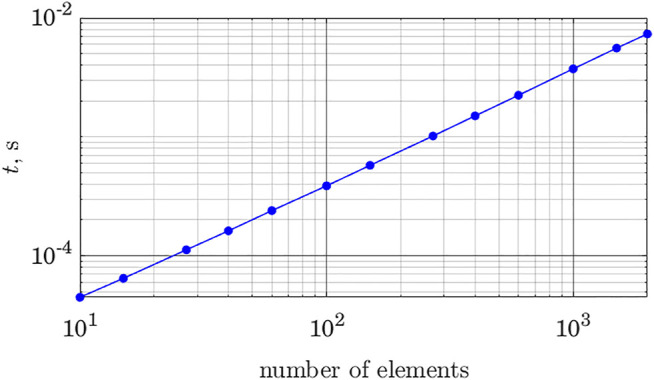
Computational time as a function of dataset size; both axes are in logarithmic scale.

Another source of growth in the computational time relates to the dataset search. Algorithm 2 requires checking ellipsoids found by the solver against each entry in the dataset, making the number of checks proportional to the dataset size. Figure 6 demonstrates how the computational time depends on the number of entries in the dataset. The time cost is computed for the algorithm run on a PC with Intel i7 9700K processor, with 32 Gb memory and the dataset stored on an SSD.

The graph was produced by running the algorithm 10^3^ times and taking an average, displayed as the data point. As implied by Algorithm 2, the number of operations (matrix multiplications and computation of logarithms) depends linearly on the number of entries in the dataset.

## 9 Conclusion

In this paper we presented a method for deformation-aware trajectory planning for deformable tensegrity drones. Employing two conservative assumptions (over-approximating the shape of tensegrity drone as an ellipsoid, and assuming that in order to pass between two regions, the drone needs to pass through their intersection) the proposed method takes advantage of the convexity of the resulting problem, as well as of the possibility to separate it into a number of independent optimization problems, avoiding the growth in the number of decision variables with the increase in the size of the path to be planned. Pre-computing Löwner-John ellipsoids and their eigendecomposition allow to keeping computational cost of the algorithm relatively low, with the linear growth in the number of elements in the dataset.

## Data Availability

The original contributions presented in the study are included in the article/Supplementary Material, further inquiries can be directed to the corresponding author.
